# RESULTS OF A STEPPED EXERCISE PROGRAM AMONG VETERANS WITH KNEE OSTEOARTHRITIS AND CO-OCCURRING BACK PAIN

**DOI:** 10.1093/geroni/igae098.4068

**Published:** 2024-12-31

**Authors:** Brett Burrows, Sandra Woolson, Cynthia Coffman, Kelli Allen

**Affiliations:** Duke University School of Medicine, Durham, North Carolina, United States; Durham VA Health Care System, Durham, North Carolina, United States; Durham VA Health Care System, Durham, North Carolina, United States; Durham VA Health Care System, Durham, North Carolina, United States

## Abstract

Co-occurring back pain can exacerbate an already high risk of disability among patients with knee osteoarthritis (OA). We described the effect of a stepped exercise program for patients with knee OA (STEP-KOA) versus arthritis education (AE) on Western Ontario and McMaster Universities Osteoarthritis Index (WOMAC) scores for participants with and without co-occurring back pain. Veterans with knee OA (n= 345) were randomized 2:1 to STEP-KOA or AE control group that received mailed materials. STEP-KOA began with 3 months of internet-supported home exercise (Step 1), followed by 3 months of bi-weekly physical activity coaching (Step 2), then 3 months of physical therapy (Step 3) if participants did not make clinically relevant improvement in pain or function with the prior step. WOMAC, which assesses lower-extremity pain, stiffness and function was the primary outcome at 9 months. Self-reported back pain was reported at baseline. Differences in mean WOMAC scores at 9-months were compared between arms in those with and without back pain using t-tests. Overall, 72.4% (n=250) reported having co-occurring back pain. Among participants with co-occurring back pain (n= 176), 9-month mean WOMAC score was 10.1 points lower (95% CI -15.5, -4.7; p< 0.001) in STEP-KOA vs. AE. Among participants without co-occurring back pain (n= 79), 9-month mean WOMAC score did not differ between arms (Mean Difference= 4.3; 95% CI -5.3, 13.9; p=0.37). In conclusion, among participants with co-occurring back pain, the STEP-KOA intervention resulted in a significant reduction in mean WOMAC score at 9-months compared to AE alone.

